# Editorial: Refugees and migrants health: expanding the findings of the WHO Global Evidence Review on Health and Migration (GEHM) and beyond

**DOI:** 10.3389/fpubh.2024.1391064

**Published:** 2024-03-15

**Authors:** Palmira Immordino

**Affiliations:** Department of Health Promotion, Mother and Child Care, Internal Medicine and Medical Specialties, University Center “Migrare”, University of Palermo, Palermo, Italy

**Keywords:** migrant, refugee, healthcare, asylum seeker, health services, health policies, health determinants, inequities

The increase in the rate of displacement in the international context together with the increased vulnerability of migrant and refugee populations, makes it urgent to fill the research gaps identified in the Global Evidence Review on Health and Migration series developed by the Department of Health and Migration of the World Health Organization (WHO). This Research Topic provides a compendium of experiences, practices and protocols that attempt to address some of these research gaps. This editorial synthesizes key findings from the 11 accepted articles.

A study by Marchetti et al. aimed to understand the health needs and perceptions of health care among migrants, asylum seekers, and refugees in an Italian health care context. Findings highlighted the complex health needs of these population groups, which include mental, sexual, reproductive, and nutritional health, alongside a desire for better knowledge of the health system and integration. Recommendations for policymakers include the reorganization of health services to meet the needs of migrants, asylum seekers, and refugees, cultural mediation, peer education, and training of health professionals to improve accessibility, equity, and effectiveness.

Three papers investigated the health of refugees and migrants in the context of the COVID-19 pandemic. The scoping review by El Arab, Somerville et al. tried to collect the available evidence on the impact of COVID-19 on physical and mental wellbeing. The results showed that the vulnerabilities of these population groups was further exacerbated by COVID-19, particularly because of a lack of resources, support and adequate information. Misinformation and a lack of trust in health workers and health systems also contributed to limited access to care or preventive programs, for instance, vaccination programs. Moreover, the transition to digital tools has presented new challenges, not only related to language difficulties or a lack of technical expertise but also due to systemic issues, like the need for a bank ID, which frequently remains out of reach for these communities. This element was also examined by Wu et al. In their study, the authors explored how the pandemic impacted access to services for asylum seekers and how community-based programs in Canada responded to the challenges arising from the public health guidelines that were put in place. Their qualitative analysis showed that, on the one hand, the transition from in-person to online services created new challenges related to technological, linguistic, and material barriers, and a lack of trust related to privacy protection and security; on the other hand, organizations adapted to public health regulations by building new partnerships and collaborations to ensure the provision of services.

Brandt et al. reported the results of a cross-sectional study of adult asylum seekers arriving in Berlin to explore flight-associated risk factors for COVID-19 infection. The authors highlighted a higher likelihood of seropositivity in women, which was however reduced by frequent hygiene practices. Accommodation in a refugee shelter and poor hygiene behaviors were found to be associated with an increased risk of infection. Other associated factors were lower educational level, traveling with children or on foot, and seeking information about COVID-19.

Migrants face significant health challenges, including a high prevalence of non-communicable diseases, particularly in low- and middle-income countries. The study by Rada and Cabieses reviewed the scientific literature on hypertension prevention and control among migrants in Latin America, examining the impact of migration and health policies on access to care. It identified barriers like insurance coverage, language differences, and financial constraints. The findings emphasize the need for culturally sensitive health interventions that take into account the unique vulnerabilities, lifestyles, and gender-specific needs of migrants.

Three of these manuscripts proposed study protocols and methods.

The study by El Arab, Urbanavice et al. presented a mixed-methods study involving qualitative interviews with asylum seekers and Ukrainian refugees. The objective was to establish guidance for improving access to health services that encompassed both the prevention and management of chronic and acute conditions, through primary and secondary health care, with the aim of reducing worse health outcomes in these population groups. Furthermore, the results sought to offer a perspective on their integration within the community, including their employment and educational opportunities.

The contribution by Martinez-Donate et al. described the protocol of the “Migrante” project, a 14-year-long project that is attempting to address the health needs of Mexican migrants traveling across the Mexico-United States border region. The relevance of this study is clearly related to the population-level health data for this group, which is particularly hard to obtain. The findings will also lay the groundwork for a future long-term expansion of this migrant health observatory to shed light on the effects of migration policies on migrant health.

Finally, the study by Banaschak et al. proposed a multimodal information campaign aimed at increasing the use of medical rehabilitation by migrant children and adolescents and ultimately reducing disparities in health care. The campaign adopted a participatory approach by training individuals from migrant communities in Berlin and Hamburg as transcultural health mediators. These mediators educated other families in their native language about chronic conditions and medical rehabilitation and helped them apply for rehabilitation services.

Research on the healthy migrant effect often finds migrants in better health than their native counterparts, but this does not consistently apply to cognitive functioning in aging populations. In their article, Abuladze et al. compared the cognitive performance of the middle-aged and older foreign-born population in Estonia with the host and native populations. The authors found that Russian migrants in Estonia showed higher odds of cognitive impairment in immediate recall than Estonians, but not in verbal fluency, and that these differences were consistent with those in Russia when adjusted for age at migration. The findings challenge the healthy migrant effect, suggesting that migration impacts certain cognitive abilities differently and that age at migration plays a significant role in cognitive health outcomes.

Another study by Chen et al. focused on mental health disorders, particularly their association with poor labor market outcomes. This study found that common mental disorders and multimorbidity increased the likelihood of receiving disability pension and unemployment benefits, with variations between refugees and Swedish-born individuals. Particularly, refugees were at higher risk of unemployment when multimorbidity was present. Schizophrenia and behavioral syndromes were identified as having a particularly high risk of labor market marginalization. The findings suggest that interventions should consider the specific needs of young adults, taking into account their mental health status and refugee background.

Another work addressing the social determinants of health focused on Chinese migrant workers. The research by Yang et al. aimed to examine the link between social support and the quality of life among Chinese migrant workers, investigating how a healthy lifestyle mediates this relationship. Findings indicate that migrant workers scored higher on social support and quality of life than urban workers. Social support was positively related to quality of life for both groups, with healthy lifestyle habits mediating this relationship for migrant workers. The study highlighted the importance of enhancing social support and promoting healthy lifestyles to boost the quality of life of migrant workers.

While this Research Topic of papers does not cover all topics, it demonstrates that impactful and insightful research that contributes to policy, practice, and global knowledge in the field of health and migration, is being conducted globally and can guide policy and programmatic efforts to improve the health of refugees and migrants in their communities of origin, transit, and destination.

## Author contributions

PI: Conceptualization, Writing – original draft.

